# Boston Medical and Surgical Journal

**Published:** 1840-06

**Authors:** 


### BOSTON MEDICAL AND SURGICAL JOURNAL—PROFESSOR GROSS.

Our able and respected contemporary, in noticing the appointment of Dr. Gross to the chair of Surgery in the Medical Institute of Louisville, indulges in a remark which shows that the editor has been prejudiced by a one-sided report of the circumstances which brought about the resignation of the late Professor of Surgery. The editor of the Boston Medical and Surgical Journal, we are sure, would not willingly be instrumental in propagating a false impression with regard to his brethren. We assure him that he has been misled, and that, if he will inform himself as to the facts of the case, he will find no cause for the offensive reflection contained in the following notice:

"*Dr. Gross.*—It has been officially announced that Samuel D. Gross, M. D., was elected, at a late meeting of the trustees, professor of Surgery in the Louisville Medical Institute, to the chair vacated by the resignation of Dr. Flint. A personal acquaintance with Dr. Gross, together with an intimate knowledge of his writings, constrain us to say that the Louisville School has been exceedingly fortunate in securing this gentleman's sevices. May he never be assailed by the spirit of envy, or brow-beaten by those who suppose that talents of a high order are dangerous to the weal of those less gifted. Through the pages of his great work on Pathological Anatomy, now circulating through the land, those who are curious to know the claims of Dr. Gross to the confidence of those who control the destiny of the Louisville Institute, will find ample evidence of his fitness for the station to which he has been unanimously called." Y.

